# Nutrigenomic effects of glucosinolates on liver, muscle and distal kidney in parasite-free and salmon louse infected Atlantic salmon

**DOI:** 10.1186/s13071-016-1921-7

**Published:** 2016-12-12

**Authors:** Stanko Skugor, Helle Jodaa Holm, Anne Kari Bjelland, Jorge Pino, Øystein Evensen, Aleksei Krasnov, Simon Wadsworth

**Affiliations:** 1 Cargill Innovation Center, Sea Lice Research Centre, Oslo, Norway; 2 Norwegian University of Life Sciences, Faculty of Veterinary Medicine and Biosciences, Sea Lice Research Centre, Oslo, Norway; 3 Cargill Innovation Center, Dirdal, Norway; 4 Cargill Innovation Center, Puerto Montt, Chile; 5 Nofima AS, Ås, Norway

**Keywords:** Atlantic salmon, *Salmo salar*, Salmon louse, *Lepeophtheirus salmonis*, Glucosinolates, Functional feeds, Iron, Antioxidant, Detoxification

## Abstract

**Background:**

Reduction of *Lepeophtheirus salmonis* infection in Atlantic salmon achieved by glucosinolates (GLs) from *Brassica* plants was recently reported. However, wider application of functional feeds based on GLs requires better knowledge of their positive and adverse effects.

**Methods:**

Liver, distal kidney and muscle transcriptomes of salmon exposed to the extreme dose of GLs were profiled by microarray, while qPCR analysis followed up selected hepatic and renal responses under the extreme and moderate GLs dose during the *L. salmonis* challenge. Transcriptional analysis were complemented with measurements of organ indices, liver steatosis and plasma profiling, including indicators of cytolysis and bilirubin. Finally, the third trial was performed to quantify the effect of lower GLs doses on growth.

**Results:**

The extreme GLs dose caused a decrease in hepatic fat deposition and growth, in line with microarray findings, which suggested tissue remodeling and reduction of cellular proliferation in the skeletal muscle and liver. Lower GLs inclusion levels in a follow-up trial did not show negative effects on growth. Microarray analysis of the distal kidney pointed to activation of anti-fibrotic responses under the overexposure. However, analyses of ALT, CK and AST enzymes in plasma provided no evidence of increased cytolysis and organ damage. Prevalent activation of phase-2 detoxification genes that occurred in all three tissues could be considered part of beneficial effects caused by the extreme dose of GLs. In addition, transcriptomic evidence suggested GLs-mediated iron and heme withdrawal response, including increased heme degradation in muscle (upregulation of *heme oxygenase-1*), decrease of its synthesis in liver (downregulation of *porphobilinogen deaminase*) and increased iron sequestration from blood (hepatic induction of *hepcidin-1* and renal induction of intracellular storage protein *ferritin*). This response could be advantageous for salmon upon encountering lice, which depend on the host for the provision of iron carrying heme. Most of the hepatic genes studied by qPCR showed similar expression levels in fish exposed to GLs, lice and their combination, while renal induction of *leptin* suggested heightened stress by the combination of extreme dose of GLs and lice. High expression of* interferon* *γ* (cytokine considered organ-protective in mammalian kidney) was detected at the moderate GLs level. This fish also showed highest plasma bilirubin levels (degradation product of heme), and had lowest number of attached lice, further supporting hypothesis that making heme unavailable to lice could be part of an effective anti-parasitic strategy.

**Conclusions:**

Modulation of detoxification and iron metabolism in Atlantic salmon tissues could be beneficial prior and during lice infestations. Investigation of anti-lice functional feeds based on low and moderate GLs inclusion levels thus deserves further attention.

**Electronic supplementary material:**

The online version of this article (doi:10.1186/s13071-016-1921-7) contains supplementary material, which is available to authorized users.

## Background

Despite significant attention given to finding alternative control strategies of the ectoparasite salmon louse *Lepeophtheirus salmonis*, the management of infections on salmon farms still heavily relies on the use of chemical treatments [1]. Several recent studies describe the severity of the situation in detail, including incurred economic losses [2], mortalities associated with application of chemical treatments [1] and in wild salmonid populations [3–5], risk of pathogenic virus transmission [6–8], and development of resistance to available parasiticides [9–12]. As chemical treatments are becoming limited and less efficient, there is an increasing interest in the development of anti-lice functional feeds.

Protection against lice can involve modulation of inflammation at the attachment site and induction of iron regulatory mechanisms [13–16], processes that can be modulated by diet [17, 18]. GLs constitute a heterogeneous family of sulfur-rich secondary plant metabolites occurring in cruciferous plants that are grown and consumed worldwide. Upon mechanical damage, GLs are hydrolyzed by the enzyme myrosinase into compounds that defend plants against a wide range of herbivores, including insects and aquatic invertebrates (reviewed by [19]). Ingested GLs are also hydrolyzed by the intestinal microflora [20]. Isothiocyanates (ITCs) constitute the major bioactive fraction of the hydrolysis products of GLs, with antibacterial properties in vitro [21], and antifungal effector properties in live plant cells [22].

GLs and related products are toxic to parasites in direct contact, but not much is known about the mechanisms behind the avoidance behavior of chemical irritation. The ability to perceive volatile ITCs has been well documented, especially in insects [23]. Avoidance and attractant effects with non-host and host conditioned water respectively, were shown in behavioral tests in vitro, in a related, also parasitic louse species (*Caligus rogercresseyi*) [24]. An in vivo follow-up study revealed activation of putative ionotropic receptor genes that could possibly be involved in the olfaction and avoidance of salmon fed anti-lice feeds [25].

Currently, GLs and their breakdown products are attracting attention in fish nutrition research because of their parasiticidal potential against the sea louse species that infect cultured salmonids. Fish receiving GLs-based functional feeds could additionally benefit from their detoxifying and immunomodulatory properties. Modulation of cellular redox status appears to be at the core of ITCs’ bioactivity (reviewed in detail by [26]). The indirect antioxidant properties of ITCs achieved through induction of phase-2 detox enzymes are considered responsible for their anticancerogenic properties [27]. However, under certain conditions, ITCs can also induce the pro-oxidant phase-1 enzymes [26]. The positive effects on human health of GLs and ITCs present in diets rich in cruciferous vegetables were reported by several clinical studies [28–31]. However, GLs can exert anti-nutritional and toxic effects [32–36], and their wider use in aquaculture requires better knowledge of their actions. Reduced palatability and decreased growth are among the main anti-nutritional effects of overexposure to GLs/ITCs in vertebrates, (reviewed in [37]). When large quantities are ingested, the adverse health effects include deterioration of liver, kidney and thyroid function [37].

Here we report findings from several feeding trials designed to investigate both the beneficial and adverse effects of GLs, alone and during the *L. salmonis* challenge, on nutritional parameters, gene expression and physiological responses in Atlantic salmon. In Trial 1, Atlantic salmon not infected with lice (NI) but with an extreme inclusion level (13%) of the GLs-containing raw ingredient in their diet (NI-13) were compared to the control group with 0% dietary GLs (NI-C); hepatic, renal and muscle transcriptomes measured by microarrays were complemented with measurements of the growth response, liver steatosis and plasma biochemistry. Trial 2 addressed the effects of the *L. salmonis* infection and GLs on growth, plasma biochemistry, and gene expression of GLs-responsive candidates by qPCR in liver and distal kidney of infected (I) fish exposed to control feed with 0% GLs (I-C), medium (3.6%) (I-3.6) and extreme level of GLs (I-13). Finally, in Trial 3, a lower range of dietary GLs (0, 0.5, 1 and 2%) were tested against *L. salmonis* infection. Growth, hepato-somatic and intestinal-somatic indices, liver steatosis and muscle tissue composition were measured in I-C, I-0.5, I-1 and I-2 study groups.

## Results

### Fish growth

In Trial 1, no significant reduction in growth was seen in parasite free NI-13 fish at the end of the 17–18 day exposure period to the extreme dose of GLs (Table 1). After a longer exposure period (47 days), significant (ANOVA: *F*_(2,177)_ = 24.86; I-C *vs* I-3.6: *P* < 0.0001; I-C *vs* I-13: *P* < 0.0001) growth reductions were observed in Trial 2 in I-13 and I-3.6 groups, 17 and 14% lower in comparison to I-C, respectively (Table 2). The calculated condition factor (CF) was found to be very similar in NI-C and NI-13 (Table 1), but significantly (ANOVA: *F*_(2,177)_ = 11.37, I-C *vs* I-3.6 *P* < 0.0001, I-C *vs* I-13 *P* < 0.001) different in Trial 2 (Table 2). The lower inclusion levels in Trial 3 of the GLs-containing raw ingredient in I-0.5, I-1 and I-2 did not result in significant differences in weight and CF between the dietary groups (Table 3). No differences in feed consumption were found in any of the trials.

**Table 1:** Mean weight ± standard deviation (SD) and mean condition factor ± SD in not infected fish exposed to 0% of the GLs-containing raw ingredient (NI-C) and 13% (NI-13) in Trial 1. Data was analyzed by *t*-test

Trial 1	NI-C (*n* = 18)	NI-13 (*n* = 18)
Weight^a^ (g)	825.6 ± 117.5	805 ± 139
CF^b^	1.52 ± 0.08	1.56 ± 0.08

^a^t-test: t_(34)_ = 0.49, *P* = 0.62

^b^Condition factor was calculated by the formula (weight*100/length^3^) for each individual fish. t-test: t(_34_) = 1.37, *P* = 0.18

**Table 2:** Mean weight ± standard deviation (SD) and condition factor ± SD in *L. salmonis-*infected fish exposed to 0% of the GLs-containing raw ingredient (I-C), 3.6% (I-3.6) and 13% (I-13) in Trial 2. Data were analyzed by One-way ANOVA

Trial 2	I-C (*n* = 60)	I-3.6 (*n* = 60)	I-13 (*n* = 60)
Weight^a^ (g)	871 ± 127	751 ± 121^****^	726 ± 113^****^
CF^b^	1.43 ± 0.13	1.54 ± 0.16^****^	1.52 ± 0.13^***^

***P < 0.001, **** *P* < 0.0001: significant differences in comparisons with control

^a^ANOVA: *F*_(2,177)_ = 24.86, I-C *vs* I-3.6 *P* < 0.0001, I-C *vs* I-13 *P* < 0.0001

^b^Condition factor was calculated by the formula (weight*100/length^3^) for each individual fish. ANOVA: *F*_(2,177)_ = 11.37, I-C *vs* I-3.6 *P* < 0.0001, I-C *vs* I-13 *P* < 0.001

**Table 3:** Mean weight ± SD and condition factor ± SD in *L. salmonis*-infected fish exposed to 0% of the GLs-containing raw ingredient (I-C), 0.5% (I-0.5), 1% (I-1) and 2% (I-2) in Trial 3

Trial 3	I-C (*n* = 78)	I-0.5 (*n* = 76)	I-1 (*n* = 72)	I-2 (*n* = 73)
Weight^a^	540 ± 110	554 ± 103	540 ± 119	553 ± 103
CF^b^	1.2 ± 0.08	1.2 ± 0.08	1.2 ± 0.07	1.2 ± 0.05

^a^ANOVA: *F*_(3,292)_ = 0.4, *P* = 0.75

^b^Condition factor was calculated by the formula (weight*100/length^3^) for each individual fish. ANOVA: *F*_(3,291)_ = 1.85, *P* = 0.14

### Plasma profiling

**Fig. 1:**
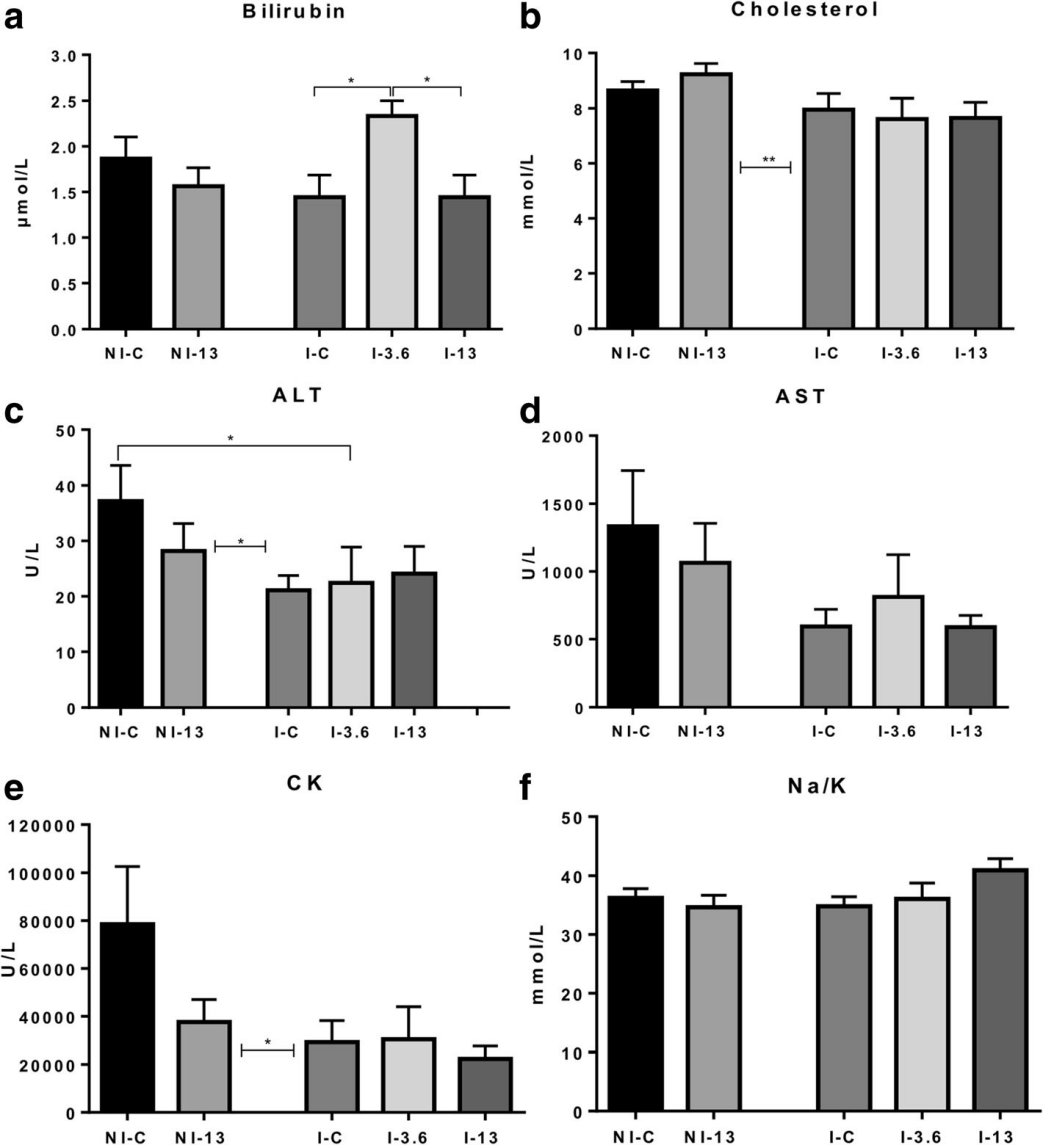
Blood plasma levels (mean ± SEM) of total bilirubin (**a**), cholesterol (**b**), alanine aminotransferase (ALT) (**c**), aspartate aminotransferase (AST) (**d**), creatine kinase (CK) (**e**) and sodium (Na^+^) to potassium (K^+^) ratio (Na/K) (**f**) in not infected (NI) fish exposed to 0% of GLs (NI-C) and an extreme dose of GLs (NI-13) and infected fish (I) fed feed with 0% of GLs-containing raw ingredient (I-C), 3.6% (I-3.6) and 13% (I-13). Blood plasma profiling was performed on 15 individuals from NI-C and 16 individuals from NI-13 in Trial 1, and 9 fish from each of the groups in Trial 2. Asterisks shown between NI and I groups refer to statistical differences of NI-C and NI-13 as one group *vs* I-C, I-3.6 and I-13 as the other group. Asterisks shown above bars denote significant differences between two groups. *P < 0.05, **P < 0.01

A basic panel of plasma tests was performed on parasite free fish from Trial 1 (NI-C and NI-13) and lice infected fish from Trial 2 (I-C, I-3.6 and I-13) (Fig. 1). The elevated levels of profiled enzymes in plasma are considered good indicators of cytolysis and cell leakage [38]. Alanine aminotransferase (ALT) and aspartate aminotransferase (AST) are found in liver parenchymal cells, and also in kidney, muscle and other tissues [38], while creatine kinase (CK) is an enzyme that mainly increases due to leakage from muscle cells [39, 40]. No differences were found in plasma levels of ALT, AST and CK between NI-C and NI-13. Interestingly, levels of all three profiled enzymes were lower in Trial 2 during the lice infection in comparison to values measured in lice free fish in Trial 1. The two trials are directly comparable as both took place under the same conditions and at about the same time. Cholesterol levels were also significantly (*t*-test: *t*_(56)_ = 2.8, *P* = 0.007) lower in infected fish than in fish not exposed to lice. Bilirubin, which is predominantly formed by the breakdown of heme present in hemoglobin [38], showed the highest level in I-3.6 and was significantly different from the level measured in I-C and I-13 (Kruskal-Wallis H-test: *χ* ^2^ = 5.99, *df* = 2; I-C *vs* I-3.6: *P* = 0.02; I-13 *vs* I-3.6: *P* = 0.05). Stress causes ionic imbalances in fish (see Djordjevic et al. [41] and references therein). We have previously seen an increase in sodium (Na^+^) ions in cortisol injected salmon during the lice challenge [42], hence we measured Na^+^ and potassium (K^+^) plasma levels and calculated their ratio (Na/K) for each group. Na^+^ decreased in NI-13 fish while K^+^ decreased in I-13, in comparison to their respective controls (data not shown), while the Na/K was found to be highest in I-13.

### Liver steatosis

**Fig. 2:**
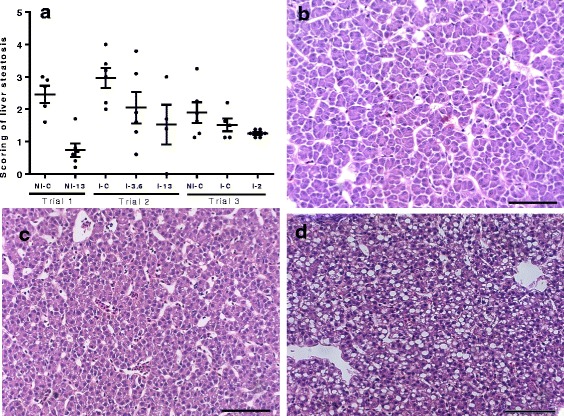
**a** Scoring of liver steatosis by light microscopy in Trial 1, 2 and 3. Liver sections from Trial 1 (NI-C and NI-13), Trial 2 (I-C, I-3.6 and I-13) and Trial 3 (NI- C, I-C and I-2) were scored from 0 to 5 [88], based on the degree of vacuolization in the cytoplasm and the degree of distribution of the vacuolated hepatocytes (Additional file 1: Table S2). 4–6 fish in each group were analyzed. *Solid black line* shows the mean score ± SEM in each group, and *black dots* show the individual fish scores. **b-d** Exemplary images of livers showing different level of steatosis. **b** Micrograph of a fish from group I-3.6 fish (Trial 2) with a score of 0. **c** Micrograph of a fish from group NI-C fish from Trial 3 with a score of 1. **d** Micrograph of a fish from group I-C fish from Trial 2 with a score of 3. *Scale-bars*: 100 μm

Liver steatosis results from the three trials are shown in Fig. 2. A lowering effect of GLs on steatosis was observed in all trials, while Trial 3 captured a smaller lowering effect of lice infection on liver steatosis.

### Organo-somatic indices and flesh quality

**Fig. 3:**
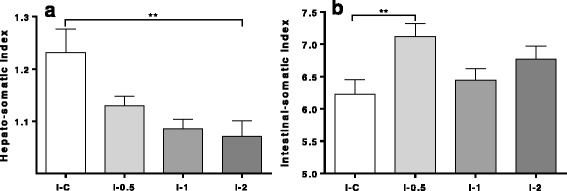
Organ indices (mean ± SEM) in lice infected fish (I) fed inclusion levels of 0% (I-C), 0.5% (I-0.5), 1% (I-1) and 2% (I-2) of GLs. **a** Hepato-somatic indices (HSI). **b** Intestinal-somatic indices (ISI). Number of fish in each group is 10. Asterisks denote level of significance between groups: **P < 0.01. ISI data was analyzed by One-way ANOVA followed by Tukey’s multiple comparisons test, while HSI data was analyzed by the Kruskal-Wallis test followed by the *post-hoc* Dunn’s test

In Trial 3, the hepato-somatic index (HSI) and intestinal-somatic index (ISI) were calculated based on measurements taken from 10 fish from each of the study groups (Fig. 3). The HSI declined as the inclusion of GLs increased, being significantly (Kruskal-Wallis H-test: *χ* ^2^ = 7.8, *df* = 3, *P* = 0.008) different between I-2 and I-C. GLs showed the opposite effect on ISI, with the significant (ANOVA: *F*_(3,37)_ = 3.8, *P* = 0.002) difference observed between I-C and I-0.5 fish.

Flesh quality parameters were determined by the near infrared spectroscopy (NIR) performed on Norwegian quality cut (NQC) samples (Table 4). Almost all of the significant changes in the fatty acid profile were observed between I-0.5 fish and I-C, and I-2 and I-C.

**Table 4:** Near infrared spectroscopy (NIR) of Norwegian quality cut (NQC) samples from Trial 3

Trial 3	14:0^a^	16:0^b^	18:1^c^	22:6n-3^d^
	(*n* = 10)	(*n* = 10)	(*n* = 10)	(*n* = 10)
I-0.5	3.23 ± 0.05^**^	15.85 ± 0.37^**^	29.18 ± 0.38	7.52 ± 0.20^**^
I-1	3.31 ± 0.07	14.94 ± 0.47	29.49 ± 0.76	7.80 ± 0.20
I-2	3.37 ± 0.06	15.82 ± 0.32^**^	28.01 ± 0.65^*^	7.78 ± 0.21^*^
I-C	5.78 ± 1.55	12.67 ± 1.34	23.47 ± 2.66	8.64 ± 0.28

Values are shown as mean ± SEM.

*P < 0.05, ** *P* < 0.01: significant differences in comparison with control

^a^14:0 Kruskal-Wallis H-test: *χ* ^2^ = 7.8, *df* = 3, *P* = 0.01

^b^16:0 Kruskal-Wallis H-test: *χ* ^2^ = 7.8, *df* = 3, *P* = 0.027; I-0.5 *vs* I-C *P* = 0.0096; I-2 *vs* I-C *P* = 0.009

^c^18:1 Kruskal-Wallis H-test: *χ* ^2^ = 7.8, *df* = 3, *P* = 0.03

^d^22:6n-3 ANOVA: *F*_(3,34)_ = 4.5; I-0.5 *vs* I-C *P* = 0.0064; I-2 *vs* I-C *P* = 0.05)

Lice infected (I) fish were fed inclusion levels of 0% GLs-containing raw ingredient (I-C), 0.5% (I-0.5), 1% (I-1) and 2% (I-2). Levels of 14:0, 16:0, 18:1, 22:6n-3 are shown, as they were the only parameters that were significantly different in the One-way ANOVA or Kruskal-Wallis test

### Microarray analyses

Microarray analyses were performed on liver, distal kidney and muscle samples from the NI-C and NI-13 fish from Trial 1. The criteria for differentially expressed genes (DEGs) were selected by comparison of the test NI-13 group to NI-C, with log2-ER > 0.6 and *P* < 0.05. Magnitude of diet-induced changes was similar in all tissues, while the number of DEGs that met our criteria was highest in the liver (232), followed by the distal kidney (188) and the muscle (156).

### Liver

Genes with roles in cell cycle and related processes (chromatin organisation regulation, DNA replication and repair) comprised a large part of differentially expressed genes (Table 5). Increased expression was shown by several genes involved in the negative regulation of cellular proliferation (e.g. *cullin 1b*, *btg1*, *c ostars family protein abracl*) while a suite of genes coding for proteins required for cell cycle, DNA replication and cellular division (e.g. *securin*, *condensin complex subunit 3* and cyclins *G2/mitotic-specific cyclin-B1* and *cyclin-A2*) were downregulated. Activation of detoxification genes from both phase-1 (oxidation, reduction, and hydrolysis reactions) and phase-2 (conjugation reactions that increase water solubility of products generated by phase-1 enzymes) pathways was observed (Table 5). Phase-1 monooxygenases *cytochrome P450 24A1* (*cyp24a1*) may oxidise either xenobiotics or endogenous compounds. GLs-enriched diet also activated *epoxide hydrolase* (*ephx*) *1* and *ephx 2* that have roles in the protection from cyclic epoxides. Phase-2 detox metabolism was represented by genes from well-known families responsive to ITCs in mammals [43–46]: *UDP Glucuronosyltransferase 1 family polypeptide b7* (*ugt1b7*), *arylamine N-acetyltransferase*, *pineal gland isozyme NAT-10* (*ary1*), involved in the detoxification of hydrazine and arylamine drugs, and *glutathione S-transferase theta* (*gstt*) *1* and *gstt3*. Stimulation of biotransformation was in line with the slight induction of stress responses witnessed by the upregulation of *glucocorticoid receptor* and *transcription factor jun b*. Expression of several other genes with important metabolic roles was affected. The leader among the induced genes was *CMP-sialic acid transporter* (Table 5) involved in the transfer of sialic acid into the Golgi lumen where its conjugation to acceptor molecules takes place. *Pyruvate dehydrogenase kinase isozyme 2* (*pdk2*) is a master regulator of metabolic fluxes through the pathways of glucose and lipid metabolism. Also of note was stimulation of *bile salt export pump*. The observed induction of *hepcidin-1* (*hepc1*) and *cytochrome b reductase 1* (*cybrd1*), the key regulators of iron uptake indicated a decrease of plasma iron levels (Table 5). *Porphobilinogen deaminase*, involved in heme biosynthesis, and *probable cytosolic iron-sulfur protein assembly protein ciao 1*, key component of the cytosolic iron-sulfur protein assembly complex, were downregulated. Dietary suppression of a handful of immune genes was observed in the NI-13 group while a few negative regulators of immune responses were found among upregulated genes (data not shown). In contrast, we observed the hepatic induction of four complement system genes (Table 5).

**Table 5:** Differentially expressed genes in the liver of not infected (NI) salmon fed an extreme dose of GLs-containing raw ingredient (NI-13) in comparison to NI salmon fed 0% dietary GLs (NI-C). Data are log2-ER

	log2-ER
Negative regulation of proliferation	
*Cullin 1b*	1.82
*Btg1*	1.66
*Costars family protein abracl*	1.61
*NOD-like receptor C*	1.42
*HIV-1 Tat interactive protein 2*	1.01
*Nitrilase homolog 2*	0.90
Positive regulation of proliferation	
*Transmembrane protein 53*	-1.17
*Cell division cycle protein 23 homolog*	-1.20
*Cyclin-A2*	-1.21
*Arntl2 protein*	-1.23
*Ornithine decarboxylase 1*	-1.29
*G2/mitotic-specific cyclin-B1*	-1.47
*Placenta-specific gene 8 protein*	-1.62
Chromatide segregation and chromosome organization	
*Haspin*	-1.03
*N-acetyltransferase esco1*	-1.20
*Securin*	-1.65
DNA replication	
*Ribonucleoside-diphosphate reductase large subunit*	-1.03
Chromatin regulation	
*Histone deacetylase 2*	-1.00
*DnaJ homolog subfamily C member 2*	-1.06
*Lamin B receptor*	-1.21
*Condensin complex subunit 3*	-1.56
DNA damage and repair	
*Uracil-DNA Glycosylase*	-1.06
*Ubiquitin carboxyl-terminal hydrolase isozyme L5*	-1.18
Biotransformation/detoxification	
*Cytochrome P450 24A1, mitochondrial precursor*	1.51
*Glucocorticoid receptor*	1.51
*Arylamine N-acetyltransferase, pineal Gland isozyme NAT-10*	1.38
*Transcription factor jun-B*	1.25
*Glutathione S-transferase 3*	1.00
*Glutathione S-transferase theta-1*	0.93
*Epoxide hydrolase 1*	0.85
*Epoxide hydrolase 2 cytoplasmic*	0.92
*UDP Glucuronosyltransferase 1 family polypeptide b7 short isoform*	0.81
Liver function	
*CMP-sialic acid transporter*	2.00
*Pyruvate dehydrogenase kinase isozyme 2, mitochondrial precursor*	1.54
*Bile salt export pump*	1.34
*Hydroxyacid-oxoacid transhydrogenase, mitochondrial precursor*	-1.08
*Novel protein similar to vertebrate scavenger receptor protein*	-1.60
Iron metabolism	
*Hepcidin-1*	1.60
*Cytochrome b reductase 1*	1.29
*Porphobilinogen deaminase*	-0.98
*Probable cytosolic iron-sulfur protein assembly protein ciao 1*	-0.92
Complement immune response	
*Complement factor H precursor*	1.33
*Properdin P factor 2*	1.04
*Properdin P factor 3*	0.94
*Complement C1q-like protein 4*	0.88

### Distal kidney

A suite of genes involved in xenobiotic metabolism were upregulated (Table 6). *NAD(P)H dehydrogenase quinone 1* (*nqo1*) is a highly-inducible gene coding for a multifunctional antioxidant enzyme that is typically coordinately regulated with other detoxifying genes responsive to ITCs [47]. Several genes that could serve as inhibitor of calcification and renal stone formation included the upregulated *fetuin-A* [48] (Table 6) and *serine-pyruvate aminotransferase, mitochondrial* with double metabolic roles, gluconeogenesis in mitochondria and peroxisomal detoxification of glyoxylate (Table 6). The latter function prevents calcium oxalate kidney stone formation [49]. *Leptin*, which was induced by diet containing GLs, may contribute to the deterioration of renal function through fostering proteinuria and TGFβ-mediated deposition of proteins in the extracellular matrix (ECM) [50] (Table 6). Multiple indications support this claim. Two inhibitors of TGFβ signalling were suppressed; *TGFβ-1-induced transcript 1 protein* that also regulates Wnt pathway and *TGFβ receptor III* that could act as a decoy receptor involved in capturing and retaining TGFβ [51]. Increased expression of *collagen a3(I)* was in parallel with downregulation of *procollagen C-endopeptidase enhancer 1* that enhances collagen degradation. Upregulation of *microfibrillar-associated protein 1*, component of the elastin-associated extracellular microfibrils and fibrinogen alpha chain could contribute to deposition of extracellular insoluble fibrils, which cause progressive renal dysfunction [52]. As could be expected, a group of genes that might play protective roles against renal fibrosis was simultaneously activated by exposure to the high dose of dietary GLs, including *ski-interacting protein* that inhibits TGFβ-mediated responses [53], *Wnt-5b* that inhibits activation of the canonical (pro-fibrotic) Wnt pathway [54] and *matrix metalloproteinase 9* involved in digestion of ECM (Table 6). We also observed regulation of a number of immune genes (data not shown), some of which have known anti-fibrotic properties [55], such as interferon γ (*ifnγ*) (Table 6) or might act as pro-fibrotic factors and contribute to tissue damage. Regulation of iron metabolism was supported by the expression of two sideroflexins and one ferritin gene (Table 6).

**Table 6:** Differentially expressed genes in distal kidney of not infected (NI) salmon fed an extreme dose of GLs-containing raw ingredient (NI-13) in comparison to NI salmon fed 0% dietary GLs (NI-C). Data are log2-ER

	log2-ER
Biotransformation, detoxification	
*Solute carrier family 22 member 2*	1.41
*NAD(P)H dehydrogenase quinone 1*	1.26
*Serine--pyruvate aminotransferase, mitochondrial precursor*	1.24
*Glutamate-cysteine ligase catalytic subunit*	0.95
*Epoxide hydrolase 2*	-0.90
Oxidation-reduction processes	
*Sarcosine dehydrogenase*	1.11
*Ubiquinol-cytochrome c reductase core I protein*	1.02
*Cytochrome B*	-0.94
Regulation of fibrosis and kidney stone formation, protection from injury	
*4-hydroxyphenylpyruvate dioxygenase*	2.43
*Deltex-3-like*	1.32
*Leptin*	1.22
*Solute carrier family 13 member 3*	1.05
*Fetuin-A*	1.03
*Interferon γ*	0.99
*Ski-interacting protein*	0.97
*Sulfide quinone reductase-like (Yeast)*	-1.08
*Serine/threonine/tyrosine-interacting protein*	-1.15
*Sparc precursor*	-1.17
*Relaxin-3*	-2.01
Extracellular matrix components and regulation	
*Fibrinogen alpha chain*	1.07
*Wnt-5b*	1.06
*Collagen a3(I)*	0.98
*Microfibrillar-associated protein 1*	0.86
*Transforming growth factor, beta (TGFβ)- receptor III*	-1.02
*Transforming growth factor beta (TGFβ)-1-induced transcript 1 protein*	-1.14
Proteolysis	
*Matrix metalloproteinase 9*	1.15
*OTU domain-containing protein 6B*	0.90
*Prepro-cathepsin C*	0.86
*Proteasome subunit alpha type-1*	0.83
*Procollagen C-endopeptidase enhancer 1*	-0.92
*N-acetylated alpha-linked acidic dipeptidase-like 1*	-1.01
*Adamts15*	-0.88
Iron homeostasis	
*Sideroflexin-2*	0.89
*Sideroflexin-4*	0.84
*Ferritin, middle subunit*	0.83

### Muscle

Proapoptotic and inhibitory effects on proliferation in the muscle tissue were inferred from upregulated genes (Table 7), including *actin-related protein 2/3 complex subunit 1B*, involved in the regulation of actin polymerization [56]; *Bax* that by antagonizing one of the apoptosis repressors accelerates programmed cell death [57], and *androgen-induced proliferation inhibitor* that plays a role in proliferative arrest [58]. Further support came from the downregulated *heparin-binding growth factor 1* that promotes cardiac hypertrophy and smooth muscle cell proliferation [59] (Table 7). Many genes with roles in diverse aspects of muscle-biology were regulated; *myosin Va* has a role in actin filament-based movement [60]; *four and a half LIM domains protein 1* [61] has a role in muscle development or hypertrophy; *sodium/hydrogen exchanger* is involved in muscle remodeling [62]; *tetranectin* is involved in muscle regeneration and muscle cell differentiation [63]; *myotubularin* plays a role in skeletal muscle maintenance [64]; and *protein arginine methyltransferase 5* is required for myogenesis and is also a positive modulator of insulin-mediated glucose uptake in skeletal muscle cells [65]. Active remodeling of intracellular structures in muscle tissue was evidenced by upregulation of *stathmin*, which disrupts the microtubule array [66] (Table 7). *Microtubule-associated protein 1 light chain 3 alpha* and *tripartite motif-containing 55b* that plays regulatory roles in the myofibril assembly [67] were induced by the GLs-enriched diet. The suppressed *aryl hydrocarbon receptor 2 beta* (*ahR2b*) indicated fine-tuning of selected phase-1 and −2 cytochrome P450 isoforms, as has been described for the *ahR2b* mammalian counterpart [68] (Table 7). *Glutathione transferase omega-1* (*gsto1*) with dual roles in Ca-mediated muscle contraction, and cellular redox homeostasis as phase-2 biotransformation enzyme [69] was also upregulated (Table 7). The gene *6-phosphogluconolactonase* (*pgls*), coding for an enzyme required for the functioning of the pentose phosphate pathway when the rate of oxidation of NADPH is accelerated [70], was also induced. Increased expression of *pgls* could contribute to decreased lifetime of 6-phosphogluconolactone, its highly reactive and potentially toxic substrate. *Alcohol dehydrogenase class-3* (*adh3*)*,* also induced, constitutes the primary defence mechanism against formaldehyde damage and may also indirectly mediate protection of proteins against oxidation [71]. Gene encoding *heme oxygenase 1* (*ho-1*) that has important antioxidant and cytoprotective activities was the most highly induced gene by dietary GLs in muscle (Table 7). Previously, it was shown that ITCs-mediated induction of heme degrading HO-1 exerts protective effects in kidney [72]. Together with activation of *uroporphyrinogen decarboxylase*, involved in heme biosynthesis, these findings suggest increased turnover of heme under GLs exposure.

**Table 7:** Differentially expressed genes in the muscle of not infected (NI) salmon fed an extreme dose of GLs-containing raw ingredient (NI-13) in comparison to NI salmon fed 0% dietary GLs (NI-C). Data are log2-ER

	log2-ER
Positive regulation of proliferation	
*Ccr4-not transcription complex subunit 6*	1.13
*Placenta-specific gene 8 protein*	0.98
*Haspin*	-1.08
Negative regulation of proliferation, apoptosis	
*Actin-related protein 2/3 complex subunit 1B*	1.35
*Kruppel-like factor 11*	1.26
*Androgen-induced proliferation inhibitor*	1.21
*Tumor necrosis factor receptor superfamily member 1A*	0.88
*Bax*	0.86
*Caspase-activated DNase*	0.82
*Cyclin-D-binding Myb-like transcription factor 1*	-0.84
DNA replication	
*Nuclear factor 1*	1.31
*DNA replication licensing factor mcm3*	-0.83
*DNA replication licensing factor mcm5*	-1.11
DNA damage and repair	
*E3 sumo-protein ligase nse2*	-0.88
*TFIIH basal transcription factor complex helicase XPB subunit*	-0.89
*FACT complex large subunit*	-1.00
*Ubiquitin-conjugating enzyme E2 T*	-1.43
Nucleotide metabolism	
*Adenylosuccinate synthetase isozyme 2*	-0.98
*Deoxycytidylate deaminase*	-1.22
Muscle metabolism, myogenesis	
*Acta1 protein*	1.26
*Microtubule-associated protein 1 light chain 3 alpha*	1.17
*Tetranectin*	1.07
*Protein arginine methyltransferase 5*	1.03
*Myosin 1*	1.00
*Tripartite motif-containing 55b*	0.84
*Four and a half LIM domains protein 1*	-0.81
*Heparin-binding growth factor 1*	-0.85
*Myosin Va*	-1.03
*Sodium/hydrogen exchanger*	-1.45
*Myotubularin*	-1.93
Negative regulation of myogenesis	
*Cardiomyopathy associated 5 like*	1.29
*Stathmin*	1.03
*Histone deacetylase 4*	-1.66
Biotranformation, detoxification	
*6-phosphogluconolactonase*	1.21
*Glutathione transferase omega-1*	1.10
*Cytochrome b-c1 complex subunit 6, mitochondrial*	1.01
*Alcohol dehydrogenase class-3*	0.84
*Aryl hydrocarbon receptor 2 beta*	-0.86
*Cytochrome P450 1A1*	-0.86
Iron metabolism	
*Heme oxygenase 1*	1.69
*Proton-coupled folate transporter*	1.29
*NADH-cytochrome b5 reductase 1*	0.94
*Uroporphyrinogen decarboxylase*	0.84
*NADPH-dependent diflavin oxidoreductase 1*	-0.96
*Sideroflexin-2*	-1.17

### qPCR analyses

**Fig. 4:**
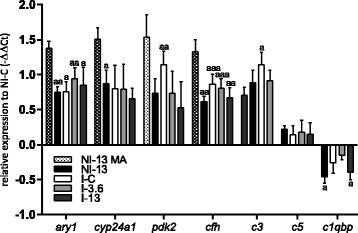
Hepatic gene expression of *ary1*, *cyp24a1* and *pdk2* with roles in metabolic adaptation to nutrient availability, complement regulator *cfh*, complement components *c3* and *c5*, and complement regulator *c1qbp* measured by qPCR and shown as mean -ΔΔCt ± SEM. The first bar for each gene shows the logER value measured by microarray in not infected (NI) fish (*n* = 5) fed 13% of the GLs-containing raw ingredient (NI-13). Gene expression in infected (I) fish fed increasing levels of GLs-containing raw ingredient, 0% (I-C), 3.6% (I-3.6) and 13% (I-13), were measured by qPCR. The zero is set to NI fish fed 0% dietary GLs (NI-C). Number of fish in each group is 9. The letter “a” denotes significant expression difference to NI-C, “aaa” when *P* < 0.001, “aa” when *P* < 0.01 and “a” when *P* < 0.05

**Fig. 5:**
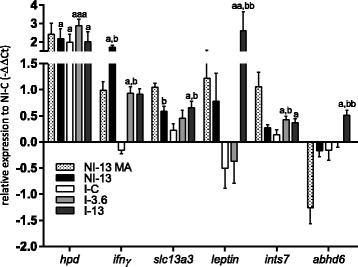
Renal gene expression of *hpd* from the tyrosine breakdown pathway, anti-fibrotic *ifnγ, slc13a3* involved in the maintenance of citrate levels, pro-fibrotic *leptin*, the DNA damage response gene *ints7*, and *abhd6* with functions in the regulation of lipogenesis in kidney, measured by qPCR and shown as mean -ΔΔCt ± SEM. The first bar for each gene shows the logER value measured by microarray in not infected (NI) fish (*n* = 5) fed feed with 13% inclusion level of GLs-containing raw ingredient (NI-13). Gene expression in infected (I) fish fed increasing levels of GLs-containing raw ingredient, 0% (I-C), 3.6% (I-3.6) and 13% (I-13), were measured by qPCR. The zero is set to NI fish fed 0% dietary GLs (NI-C). Number of fish samples in each group is 9. The letter “a” denotes significant expression difference to NI-C, and “b” denotes significant expression difference to I-C, “aaa” when *P* < 0.001, “aa/bb” when *P* < 0.01 and “a/b” when *P* < 0.05

qPCR analyses were used to validate microarray data and, in addition, compare responses of lice-challenged salmon under the low (3.6%) and extreme dose (13%) of GLs to lice free and lice infected fish given control feed. Three complement genes that were not measured by the microarray in liver were analyzed by qPCR (*complement c3 (c3)*, *complement c5 (c5)* and *complement component 1Q binding* ﻿(*c1qbp*)), as several other genes of the complement system indicated complement activation in NI-13 (Table 5). Microarray results shown next to qPCR results of NI-13 in Figs. 4, 5 (two first bars), revealed high concordance between the two platforms. *Ary1*, involved in detoxification, and *complement factor H* (*cfh*) and *c3*, involved in the complement immune response, showed similar level of activation in lice challenged fish. *Cyp24a1*, a phase-1 detoxification gene and a complement regulator *c1qbp* were significantly (*cyp24a1*: *t*-test: *t*_(15)_ = 2.2, *P* = 0.04; *c1qbp*: *t*-test: *t*_(15)_ = 2.4, *P* = 0.03) responsive to the high dose of GLs without lice (NI-13). *pdk2* was significantly induced only in I-C (*t*-test: *t*_(15)_ = 3.121, *P* = 0.007) group. Tyrosinedegrading *4-hydroxyphenylpyruvate dioxygenase* (*hpd*) was the most highly induced gene in NI-13 on the distal kidney microarray. Elevated levels of tyrosine in the absence of HPD activity are toxic to kidney [73, 74]. qPCR analyses revealed most statistically significant (*t*-test: *t*_(15)_ = 4.2, *P* = 0.0007) upregulation of *hpd* in I-3.6 salmon. I-13 group showed highest level of *solute carrier family 13 member 3-like* (*slc13a3*), while during infestation, *ifnγ* and *integrator complex subunit 7* (*ints7*) were most highly induced under the lower dose of GLs, in I-3.6 salmon. *Ifnγ*, also highly induced in NI-13, is protective against renal injury induced by arsenite by modulation of detoxification pathways [75] and experimental renal fibrosis following chemotherapeutic exposure, explained by increasing the viability of renal tubular cells [76]. *Slc13a3* is highly expressed on the basolateral membrane of proximal kidney tubule cells, contributes to heavy metal detoxification [77, 78] and is involved in the selective uptake of Krebs cycle intermediates [77]. *Ints7* plays a role in the DNA damage response pathway that typically results in cell cycle arrest [79]. qPCR analysis revealed appreciable induction of *leptin* in I-13 (6-fold compared to NI-C) while I-C and I-3.6 groups showed slight downregulation. The knockout of *abhydrolase domain-containing protein 6* (*abhd6*), which was significantly (*t*-test NI-C *vs* I-13: *t*_(15)_ = 2.1, *P* = 0.05; I-C *vs* I-13: *t*_(16)_ = 3.1, *P* = 0.0074) induced in I-13 fish, results in downregulation of genes involved in *de novo* fatty acid synthesis and lipogenesis in murine kidney [80].

## Discussion

The interest for GLs and their breakdown products in Atlantic salmon aquaculture lies in their parasiticidal potential against salmon louse, alongside beneficial effects related to the improvement of the antioxidant status and detoxification abilities. However, based on knowledge from vertebrate studies, both adverse and positive effects of dietary GLs could be anticipated. An undesired consequence, most pronounced at the extreme dietary level of GLs, was the observed reduction in growth seen in Trial 2. Microarray profiling proposed molecular players behind the reduction in growth mediated by high levels of dietary GLs. Higher mRNA levels in NI-13 in comparison to control were seen for a number of genes involved in the negative regulation of proliferation in both liver and muscle. This was in line with a number of suppressed genes with roles in the development, maintenance and hypertrophy of muscle in the transcriptome of fish under the extreme exposure to GLs. In contrast, the negative effect on growth was not shown in Trial 3, with up to 2% of the GLs-containing raw ingredient included in the feed. With respect to other potential beneficial effects of GLs, of note was the reduction of liver steatosis (Fig. 2a) measured even at a low level of GLs (I-2) and decrease of HSI in I-2 (Fig. 3a). In Trial 3, an increase in ISI with the increasing level of GLs was revealed, being highest and significant at the lowest inclusion level of GLs (Fig. 3b). Interestingly, fillet quality traits profiled by NIR for I-0.5, I-1 and I-2 fish revealed minor differences (Table 4).

Microarrays of the distal kidney of NI-13 was characterized by the concerted activation of DNA damage response genes (Table 6), and suggested activation of anti-fibrotic responses and those implied in the prevention of renal stone formation. This was not reflected in the level of plasma indicators of tissue damage (ALT, AST and CK) in Trial 1 (Fig. 1). In fact, most had lower values in NI-13 compared to NI-C. Furthermore, reduced enzyme levels in fish from Trial 2 in comparison to fish from Trial 1 were likely the reflection of lowered tissue metabolic activity during the lice challenge, possibly most affecting muscle, as judged by the drop in CK levels. Nevertheless, evidence produced with qPCR, particularly *leptin* data (Fig. 5), pointed out that I-13 fish stand an increased risk of developing renal pathophysiology in case of prolonged simultaneous exposure to high levels of the two stressors. The increase of the Na/K ratio in I-13 (Fig. 1) could be seen as a warning sign that suggested adverse alteration of the hydromineral balance in this fish in comparison to I-C. However, the high expression level of *leptin* and *abhd6* seen in I-13 (Fig. 5) completely diminished in I-3.6, and moreover, the moderate level of GLs promoted the expression of *ints7* and *ifnγ*, the latter of which has numerous documented protective roles in the mammalian kidney.

Expectedly, GLs stimulated the expression of genes involved in detoxification. In contrast to the long-held notion that GLs-derived ITCs selectively activate phase-2 while suppressing phase-1 detoxification pathways [26, 81], our data revealed activation of a diverse group of genes related to both pathways. In addition to the well-documented antioxidant properties of ITCs related to induction of phase-2 enzymes [26], their pro-oxidant properties are likely related to the simultaneous induction of phase-1 enzymes. Phase-1 detoxification is dominated by reactions involving cytochrome P450 enzymes, which are abundantly present in the endoplasmic reticulum in liver and kidney. Hepatic induction of *cyp24a1* and two epoxide hydrolases (*ephx1* and *2*) from phase-1 (Table 5) occurred in parallel with the suppression of *ephx2* in kidney (Table 6), and *cytochrome p450 1a1* and *ahR2b*, which regulates P450 enzymes, in muscle (Table 7). Activation of genes from phase-2 metabolism was prevalent in all three tissues. The extreme GLs-enriched diet induced key genes required for glutathione (GSH) based detoxification processes that result in formation of water-soluble products that can be easily excreted. *Glutamate-cysteine ligase catalytic subunit* encoding the first rate-limiting enzyme of glutathione synthesis was upregulated in distal kidney (Table 6) while glutathione transferases that catalase conjugation of GSH to xenobiotic products of phase-1 detoxification steps, were induced in liver (*gstt1* and *gstt3*) (Table 5) and muscle (*gsto1*) (Table 7). Furthermore, observed increase in the expression of *nqo1* in distal kidney (Table 6), *ary1* and *ugt1b7* in liver (Table 5), and *pgls* and *adh3* in muscle (Table 7), may all contribute to the better protection against xenobiotics in fish exposed to dietary GLs. Another potentially important effect of GLs is regulation of iron metabolism in all three tissues. Levels of bioavailable iron are determined by intestinal absorption and macrophage recycling of iron from hemoglobin. *Hepc1* is a liver peptide that modulates intestinal iron absorption and acts to attenuate iron release from tissue macrophages and hepatocytes. Hepatic induction of *hepc1* and *cybrd1* (Table 5) in fish exposed to GLs could result in increased iron sequestration in liver, thus lowering iron plasma levels. Most of the intracellular iron is used in mitochondria for heme biosynthesis or in cytoplasm for the assembly of iron-sulfur clusters that are incorporated into extra-mitochondrial iron/sulfur containing proteins. Cellular iron status determines the extent of iron-sulfur cluster assembly and thereby regulates expression of genes for iron storage, transport, and utilization. Concomitant downregulation of *porphobilinogen deaminase*, involved in heme biosynthesis provided solid indication that iron excess stimulates cytosolic FeS cluster biogenesis (Table 5). Renal induction of *ferritin*, involved in iron sequestration within cells, additionally supported possibility that the access of iron to circulation was reduced by GLs (Table 6). Furthermore, the most highly induced gene in muscle was *ho-1* (Table 7), an enzyme with the key role in degradation of heme into iron and biliverdin, which is then converted to bilirubin [82]. Iron tissue dynamics within salmonid hosts is believed to play an important role in the outcome of lice infections [16]. Coordinated and early changes in the expression of genes involved in metabolism of iron and erythropoiesis in spleen, head kidney and liver were seen in lice-infected Atlantic salmon [83, 84]. The resistant pink salmon showed highly diverse iron sequestration and homeostasis mechanisms, including an early upregulation in the head kidney of *hepc1*, *ho-1* and several genes involved in iron tissue storage and sequestering of iron from blood [16]. *Bilirubin* that is predominantly formed by breakdown of heme present in hemoglobin showed highest level in the best-protected infected group (I-3.6, Fig. 1). The observed increase in *bilirubin* was likely not caused by liver damage, as levels of ALT and AST went down during the lice infection. With respect to protection against the parasite, of note are also decreased levels of cholesterol in all infected dietary groups in comparison to NI fish. Cholesterol deprivation of lice by the infected host can limit their growth. This was also implied in our recent study where estrogen-mediated protection was associated with a regulation of skin genes involved in cholesterol metabolism, among several other potentially beneficial processes [18]. *Lepeophtheirus salmonis*, which is an obligate parasite, lacks genes required for the cholesterol biosynthesis encoded in its genome (Prof. Frank Nilsen, personal communication).

Profiling of skin of lice-infected fish from the GLs feeding trial revealed reduced number of attached lice and massive activation of antiviral responses, likely including IFN-mediated responses [17]. The type and magnitude of immune responses at the site of parasite attachment in skin [13, 15] and in internal immune organs [85] contribute to susceptibility to *L. salmonis* in Atlantic salmon. Several complement system genes responded to GLs in NI-13 – this type of immune system preconditioning by diet could be helpful upon parasite encounter.

## Conclusions

Our findings encourage future use of GLs-based feeds due to their beneficial effects on the expression of genes with detoxifying and iron-regulatory roles in multiple fish tissues. The further refinement of anti-lice functional feeds will require understanding of how the beneficial processes can be promoted to achieve protection against lice while not decreasing growth or posing any adverse effects on tissue functions.

## Methods

### Preparation of feeds and fish trials, production of feeds and copepodids

The trials were approved by the National Animal Research Authority, in line with the “European Convention for the Protection of Vertebrate Animals used for Experimental and other Scientific purposes” were performed at Ewos Innovation in Dirdal, Norway. Feeds with various inclusion levels of glucosinolates (GLs) were produced at the Ewos Innovation plant in Dirdal, Norway. The GLs were added to feeds by spraying the powdered raw ingredient onto the base pellet under vacuum conditions. Air pressure was then allowed to return to normal and the GLs-containing powder was sucked into the core of the pellet. All diets had a pellet size of 5 mm. An overview of trials, harvested samples and applied analysis methods are shown in Additional file 1: Table S1. The study groups were denoted by trial (Trial 1 and Trial 2), treatment (not infected - NI or infected - I) and the level of ingredient (0% - C, 3.6 or 13% inclusion level).

**Fig. 6:**
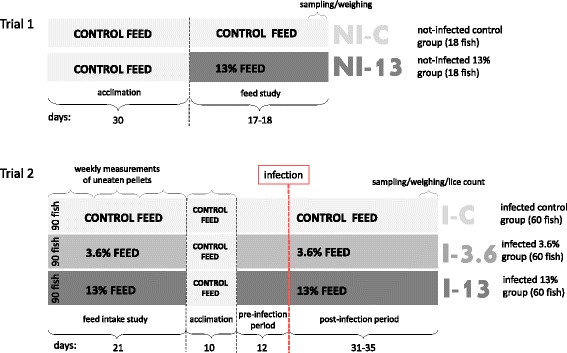
Experimental setup of Trial 1 and Trial 2 (modified from [17]). *Trial 1*. Feed study. To study responses of the feed (without infection), Atlantic salmon were fed control feed and high level (13%) of GLs-containing raw ingredient. All fish received control feed for 30 days during the acclimation period. Sampling of tissues and weighing of the fish from two groups [not-infected control group (NI-C) and not-infected 13% group (NI-13)] were performed 17–18 days after feeding experimental feeds. *Trial 2*. Feed intake and lice challenge study. Atlantic salmon were exposed to feeds containing 0, 3.6 and 13% of GLs for 21 days. Control feed was then fed for 10 days during the acclimation period. The trial continued with fish exposed to control, and 3.6 and 13% GLs feeds for 12 days (pre-infection period). The tissue sampling, weighing and lice counting from the three dietary groups [infected fish fed control feed (I-C), infected fish fed 3.6% GLs feed (I-3.6) and infected fish fed 13% GLs feed (I-13)] challenged with *L. salmonis* (50 copepodids per fish) were accomplished after 31–35 days of infection (post-infection period)

The graphical overview of Trial 1 and Trial 2 is shown in Fig. 6. Fish in six tanks with 18 fish in each were fed the 0% GLs control feed for one month (acclimation) before separation of fish into two groups (Trial 1). Fish in three tanks continued on the control feed (NI-C) while fish in three other tanks were given the 13% GLs feed (NI-13) during 17–18 days. At the end of this period weight and length measurements (*n* = 18 in each group) and samples of liver, muscle and distal kidney (*n* = 9 in each group) were taken. Tissue samples were placed in RNA*later* (Ambion®, Austin, TX, USA) and stored at 4 °C for 24 h and then stored at −80 °C until further analyses. In addition, 5 liver samples from each group were placed in neutral buffered formalin for histology. In Trial 2, fish in tank triplicates received control diet, and diets enriched with 3.6 and 13% of the GLs-containing raw ingredient during three weeks. There were 90 fish in each feed group (control, 3.6 and 13). The amount of uneaten pellets was recorded weekly to calculate the feed intake for each of the feeds over three weeks. After this, all 15 tanks were given a control feed (EWOS Opal) for 10 days, and number of fish in each group was reduced to 60. This was followed by feeding fish (*n* = 20 in each tank) control feed, 3.6 and 13% GLs enriched feeds in tank triplicates for 12 days (pre-infection period), and throughout the 31–35 day period of *L. salmonis* infection (post-infection period). During a sampling period of 4 days, number, stage and gender of lice on each fish were recorded. Furthermore, liver and distal kidney tissues were sampled in RNA*later* from 9 fish in each group. In addition, 4–6 liver samples from each group were placed in neutral buffered formalin for histology. The treatment groups tested in this part were named: infected control (I-C), infected 3.6 (I-3.6) and infected 13 (I-13). Fulton’s condition factor was calculated by the formula: (100 BWFL^−3^) [86] in both Trial 1 and Trial 2. Finally, peripheral blood from the caudal vein was collected into heparinized vacutainers from fish in each group in Trial 1 (*n* = 15–16) and Trial 2 (*n* = 9). Fish tanks used in both trials were 500 l circular flow-through tanks with an average temperature of 8.7 °C and 27.4 ppt salinity.

Trial 3 was performed in August 2013 with low inclusion levels of the GLs-containing raw ingredient (0, 0.5, 1 and 2%). Groups in this trial were thus infected control (I-C), infected 0.5% (I-0.5), infected 1% (I-1) and infected 2% (I-2) and each group of fish was allocated into three tank replicates with 23–26 fish in each tank. Tank conditions, feeding regime and infection of fish with copepodids were similar to the infection study in Trial 1 and 2 [17], but fish were fed the experimental diets for 23 days before lice challenge, and sampling was performed after 26–28 days of infection. At this time point, Norwegian Quality Cut (NQC) samples were harvested from 10 fish from each group according to the standard procedure. Liver samples (4–6) from each group were placed in neutral buffered formalin for histology. In addition, hepato-somatic and intestinal-somatic indices were calculated from 10 fish from each group, performed by weighing the liver and intestinal mass of each fish respectively and dividing this value by the fish weight.

Statistical differences of weights, CFs and organosomatic indices were assessed by One-way ANOVA with subsequent Tukey's multiple comparisons test in the GraphPad Prism Software 6.0 as criteria for Gaussian distribution were met by Shapiro-Wilkes test and in addition the equal variance test (Brown-Forsythe). Students *t*-test was used for analyzing weights and CF in Trial 1. The level of significance for all analyses was set at *P* < 0.05.

### Challenge with salmon lice

Challenge trial were performed as described in [17]. In short, the lice population used in Trial 2 and 3 originated from a nearby location (Oltesvik), and were maintained on Atlantic salmon hosts kept in 850 l circular flow tanks at the Ewos Innovation salmon lice lab. Before infection, the copepodid density was measured in a zooplankton counting chamber, where at least 4 samples were counted to ensure accuracy of estimation. Experimental infection was performed with 50 copepodids per fish. During the tissue sampling period of 4 days, when most lice had reached the preadult stages, recordings of the exact number, stage and gender of lice on each fish were made.

### RNA extraction and gene expression analysis

#### RNA extraction

Nine tissues samples of approximately 5 × 5 mm in size were excised from the fish and put in RNAlater at 4 °C and then to 80 °C until further use. Total RNA extraction was performed by the RNeasy Mini Kit (Qiagen) and Trizol (GIBCO, Life Technologies). In brief, Trizol (GIBCO, Life Technologies, Carlsbad, CA, USA), zirconium oxide beads (VWR, Oslo, Norway) and approximately 50 mg of tissue was homogenized in FastPrep-24 homogenizer (MP Biomedicals, Santa Ana, CA, USA). Chloroform was added to separate RNA into the supernatant, which was subsequently run through the RNAeasy Mini Kit clean-up procedure provided by Qiagen (Hilden, Germany). The RNA was diluted with 30 μl of RNAse free H_2_O, concentration was determined by spectrophotometry using NanoDrop ND1000 (Nanodrop Technologies, Wilmington, DE, USA) and stored at -80 °C. Integrity of RNA was assessed with Agilent 2100 BioAnalyzer (Agilent, Santa Clara, CA, US) and RNA Nano kits, and only samples with RNA integrity number (RIN) of 8 or higher were used for microarray.

#### Microarray analyses

All reagents used in the microarray procedure were from Agilent Technologies. Liver, distal kidney and muscle samples (*n* = 5) from fish from each group (NI-C, NI-13) in Trial 1 were analyzed by microarray, and compared to pooled controls of two fish from each diet from the same organ. The test samples and pooled controls were labelled with respectively Cy5 and Cy3, 100 ng of RNA per reaction, by using The Two-colour Quick Amp Labelling kits and Gene Expression Hybridization kits. The hybridization step lasted 17 h at 65 °C with rotation speed 10 rpm, followed by immersion for one minute each in Gene Expression Wash Buffer I at room temperature and subsequently washing in Gene Expression Wash Buffer II at 37 °C. By scanning slides using GenePix Personal 4100A scanner with 5 um resolution and manually adjusted laser power, an equal intensity ratio between Cy3 and Cy5 channels with minimal oversaturation was achieved. GenePix pro software 6.0 was used for feature extraction, assessment of spot quality, and spot-grid alignment. Low quality spots were flagged by the software, and Lowess normalization of log2-expression ratios (ER) was performed. Differentially expressed genes (DEGs) were selected by comparison with not infected control (NI-C): log2-ER > 0.6 and *P* < 0.05 in at least one group. Nofima’s bioinformatics system (STARS) [87] was used for data analyses.

#### cDNA synthesis and qPCR protocol

For qPCR analysis, RNA from 9 fish from the Trial 1 groups; NI-C, NI-13, and Trial 2 groups; I-3.6, I-13 and I-C, were used. NI-C from Trial 1 was deemed as an appropriate control for fish in Trial 2 as both trials took place under the same environmental conditions in the Salmon Lice Lab in Dirdal and with negligible time difference; less than a week passed between the two samplings. RNA was reverse transcribed to cDNA by using the cDNA Affinity Script (Agilent Technologies, Matriks AS, Oslo, Norway) and protocol provided by the manufacturer. Each reaction consisted of 3 μg RNA, 1 μl of random primers and 2 μl of oligo DT primers. The synthesized cDNA was diluted 10 times and stored at -20 °C until further use. The qPCR reactions were run in duplicates. Each reaction (12 μl) contained 4 μl of cDNA, 10 μM primers and SYBR Green I Master mix (Roche); analyses were run in LightCycler 480 in 96 well plates. Published gene sequences were used to design primers (Additional file 1: Table S3) for quantitative Real Time PCR (qPCR) reactions by CLC Workbench software. Cycling conditions in LightCycler 480 instrument (Roche, Applied Science) were 5 min denaturation step at 95 °C, 40 cycles of denaturation (10 s at 95 °C), annealing (20 s at 60 °C) and extension (15 s at 72 °C), followed by melting curve analysis with measurements of the fluorescence was performed in the temperature range between 65 and 97 °C. The crossing point value was found by using the maximum-second-derivative method (Roche diagnostics), followed by the -ΔΔCt method with comparison to reference gene *elongation factor 1 alpha* (*ef1α*) to find the relative expression of target genes. No signs of gDNA contamination were found by running a subset of RNA samples together with *ef1a* and SybrGreen. Specificity and efficiency were confirmed by melting curve analysis, agarose gel electrophoresis and two-fold serial dilutions of cDNA for each primer pair in triplicates. PCR efficiency of all genes ranged from 1.96 to 2. Data were analyzed with students *t*-test in the GraphPad Prism Software 6.0 if criteria for Gaussian distribution were met by Shapiro-Wilkes test. All qPCR data showed equal variance by the Brown-Forsythe test. If criteria for normality were not met, the Mann-Whitney test was used. The level of significance for all analyses was set at *P* < 0.05.

### Histology

Four to six fish selected randomly from each group in Trial 1; NI-C and NI-13, Trial 2; I-C, I-3.6 and I-13, and Trial 3; I-2 group, NI-C and I-C were subjected to histological analysis. Liver samples were fixed in neutral buffered formalin for 48 h with the change of formalin after 24 h followed by dehydration, paraffin-embedding, sectioning and haematoxylin and eosin (HE) staining by standard histological procedures. The 4 μm blinded sections were examined with a Leica DFC 420 microscope equipped with a digital imaging system (Leica Image Analysis). Steatosis scoring was performed by studying five representative fields at 20× original magnifications, selected randomly. The areas were scored for microvesicular and macrovesicular steatosis following the scoring system and method described in [88] and in Additional file 1: Table S2. A proportion of the liver samples were also stained with Periodic-Acid Schiff (PAS) to exclude glycogen accumulation as a cause of vacuole formation.

### Blood plasma profiling

Blood plasma profiling was performed on 15 individuals from NI-C and 16 individuals from NI-13 in Trial 1, and 9 fish from each of the groups in Trial 2. The full automatic Adria 1800 system in the Central clinical laboratory at the Norwegian University of Life Sciences was used to measure a basic panel of plasma parameters, including ALT (alanine aminotransferase), AST (Aspartate aminotransferase), CK (Creatine Kinase), Cholesterol, Na (sodium), K (potassium) and bilirubin. All parameters except the ions were found by measuring the optical density at a given absorbance. Na and K levels were assessed by the indirect potentiometric procedure. Significant differences for each plasma parameter were analyzed between Trial 1 and Trial 2, treating the groups in each trial as one. Significant differences between were also assessed between the groups in Trial 1 and Trial 2, separately. Data were analyzed by Student’s *t*-test or One-way ANOVA with subsequent Tukey’s multiple comparisons test in the GraphPad Prism Software 6.0 if criteria for Gaussian distribution were met by Shapiro-Wilkes test. All parameters showed equal variance in Brown-Forsythe test. If criteria for normality were not met, the Mann-Whitney or the Kruskal-Wallis test was used followed by the *post-hoc* Dunn’s test. The level of significance for all analyses was set at *P* < 0.05.

### Near infrared spectroscopy

Near infrared spectroscopy (NIR) is a spectral method based on the fact that different feed components have characteristic NIR absorption bands when exposed to specific wavelengths of infrared light. NIR analysis was performed on NQC samples from 10 individuals from each group from Trial 3 by using the NIR XDS system (Foss, Hillerød, Denmark) at Ewos Innovation, Dirdal. NIR calibration equations were found beforehand by analyzing 1300 NQC samples of fish ranging in size from 0.1 to 6 kg. Reference values were based on well-established internal and external sources. Individual NQC samples were thoroughly grinded in a meat grinder shortly after slaughter. The groups thus analyzed were I-0.5, I-1 and I-2 in addition to I-C. The levels of ash, energy, fat, moisture, phosphorous, protein, pigment, total monosaturated fatty acids, and a range of fatty acids (PUFA) including: total n-3 polyunsaturated fatty acids (PUFA), Total n-6PUFA, total PUFA, 14:0, 16:0, 16:1, 18:0, 18:1, 18:2n-6, 18:3n-3, 18:3n-6, 18:4n-3, 20:1, 20:3n-3, 20:3n-6, 20:4n-3, 20:4n-6, 20:5n-3, 22:1, 22:4n-6, 22:5n-3, 22:5n-6, 22:6n-3 were analyzed. Statistical differences were assessed by One-way ANOVA with subsequent Tukey’s multiple comparisons test in the GraphPad Prism Software 6.0 if criteria for Gaussian distribution were met by the Shapiro-Wilkes test and in addition the equal variance test (Brown-Forsythe). If criteria for normality were not met, the Kruskal-Wallis test was used followed by the *post-hoc* Dunn’s test. The level of significance for all analyses was set at *P* < 0.05.

## Additional file

Additional file 1: Table S1. An overview of samples and analysis methods applied in Trial 1, 2 and 3. **Table S2**. Scoring of liver steatosis in individual sections based on [88]. **Table S3**. Primer list. (DOCX 20 kb)

## Abbreviations

*abhd6*: *Abhydrolase domain-containing protein* 6
*adh3*: *Alcohol dehydrogenase class-3*
*ahr2b*: *Aryl hydrocarbon receptor 2 beta*
ALT: Alanine aminotransferase
*ary1*: *Arylamine N-acetyltransferase, pineal gland isozyme NAT-10*
AST: Aspartate aminotransferase
*c1qbp*: *Complement component 1Q binding*
*c3*: *Complement 3*
*c5*: *Complement 5*
*cfh*: *Complement factor H*
*ciao1*: *Probable cytosolic iron-sulfur protein assembly protein ciao 1*
CK: Creatinine kinase
*cybrd1*: Cytochrome b reductase 1
*cyp24a1*: *Cytochrome P450 24A1*
DEGs: Differentially expressed genes
ECM: Extracellular matrix
*ephx*: *Epoxide hydrolase*
GLs: Glucosinolates
*gsto1*: *Glutathione transferase omega-1*
*gstt*: *Glutathione S-transferase theta*
HSI: Hepato-somatic index
*ho-1*: *Heme oxygenase 1*
*hpd*: *Tyrosine-degrading 4-hydroxyphenylpyruvate dioxygenase*
I-0.5: Infected control fed 0.5% inclusion level of GLs
I-1: Infected control fed 1% inclusion level of GLs
I-2: Infected control fed 2% inclusion level of GLs
I-3.6: Infected control fed 3.6% inclusion level of GLs
I-13: Infected control fed 13% inclusion level of GLs
I-C: Infected control
*ifnγ*: *Interferon gamma*
*ints7*: *Integrator complex subunit 7*
ISI: Intestinal-somatic index
ITCs: *Isothiocyanate*
K: Potassium
Log2-ER: Log2-Expression Ratios
MA: Microarray
Na: Sodium
NI-13: Not infected fed 13% inclusion level of GL
NI-C: Not infected control
NIR: Near infrared spectroscopy
NQC: Norwegian quality cut
*nqo1*: *NAD(P)H dehydrogenase quinone 1*
*pdk2*: *Pyruvate dehydrogenase kinase isozyme 2*
*pgls*: *6-phosphogluconolactonase*
qPCR: Quantitative PCR
SEM: Standard error of the mean
*slc13a3*: *Solute carrier family 13 member 3-like*
*tgfβ*: *Transforming growth factor beta*
*ugt1b7*: *UDP Glucuronosyltransferase 1 family polypeptide b7*
