# Supplementary material for: Nutrigenomic effects of glucosinolates on liver, muscle and distal kidney in parasite-free and salmon louse infected Atlantic salmon
Source: Parasit Vectors. 2016 Dec 12;9:639. doi: 10.1186/s13071-016-1921-7 (PMC5153675; doi:10.1186/s13071-016-1921-7)
Supplement: Additional file 1: Table S1. — An overview of samples and analysis methods applied in Trial 1, 2 and 3. Table S2. Scoring of liver steatosis in individual sections based on [88]. Table S3. Primer list. (DOCX 20 kb) [file 13071_2016_1921_MOESM1_ESM.docx]

**Additional file 1**

**Table S1.** An overview over the various samples and analysis methods applied in Trial 1, 2 and 3. Blank spaces indicates that the analysis were not included in the trial.

| **Trial/analysis method** | **Trial 1** | **Trial 2** | **Trial 3** |
| --- | --- | --- | --- |
| **Groups** | Not infected control (NI-C)  Not infected 13 % (NI-13) | Infected control (I-C)  Infected 3.6 % (I-3.6)  Infected 13 % (I-13) | Infected control (I-C)  Infected 0.5 % (I-0.5)  Infected 1 % (I-1)  Infected 2 % (I-2) |
| **Microarray** | Liver, distal kidney, muscle |  |  |
| **qPCR** | Liver, distal kidney | Liver, distal kidney |  |
| **Weights/condition factor** | Yes | Yes | Yes |
| **Organo-somatic indices** |  |  | Hepato-somatic index (HSI)  Intestinal-somatic index (ISI) |
| **Liver steatosis scoring** | Yes | Yes | Yes |
| **Near infrared spectroscopy (NIR)** |  |  | Norwegian quality cut (NQC) |
| **Biochemical and enzyme plasma profiling** | Yes | Yes |  |

**Table S2.** Scoring of liver steatosis in individual sections based on the following system described in Martinez-Rubio *et al*. 2013 [88]

0 Formation of vacuoles in the cytoplasm, involving less than 10% of the hepatocytes and including less than 25% of the area of the individual hepatocytes

1 Formation of vacuoles in the cytoplasm, involving less than 25% of the hepatocytes and including less than 25% of the area of the individual hepatocytes

2 Formation of vacuoles in the cytoplasm, involving less than 50% of the hepatocytes and including less than 50% of the area of the individual hepatocytes

3 Formation of vacuoles in the cytoplasm, involving less than 75% of the hepatocytes and including less than 75% of the area of the individual hepatocytes

4 Formation of vacuoles in the cytoplasm, involving less than 90% of the hepatocytes and including less than 80% of the area of the individual hepatocytes

5 Formation of vacuoles in the cytoplasm, involving more than 90% of the hepatocytes and including more than 80% of the area of the individual hepatocytes

**Table S3.** Primers used for qPCR analyses

| **Gene name and symbol** | **Accession** | **Size** | **Primers** |
| --- | --- | --- | --- |
| *Arylamine N-acetyltransferase* (*ary1*) | BT046633.1 | 75 | F:GCTTGGGTGCTAAAAGAGA |
|  |  |  | R:CTGGTTGATGGTGTTGTTGT |
| *Abhydrolase-domain containing protein 6* (*abhd6*) | NM001140355.1 | 112 | F:ATCCCTCTGATCCCCTCTAC |
|  |  |  | R:CTCGAACATCCACCAATCCC |
| *Complement factor H-like* (*cfh*) | XM014123545.1 | 119 | F:TGCCGAACATAAGGATCACA |
|  |  |  | R:ATTGGCAATGAGGCAAGTTC |
| *Complement component 1Q binding* (*c1qbp*) | CA387557 | 217 | F:CGGTCTCTCTGGATGATGAGCCATA |
|  |  |  | R:CCACATCCACACGACACAGGAGTA |
| *Cytochrome P450 24A1* (*cyp24a1*) | BT059557.1 | 78 | F:ACATCTACCGCCACAGTCA |
|  |  |  | R:TCTCCACTCCTCCGATCT |
| *Complement c3* (*c3*) | L24433.1 | 106 | F:GAGGAAAGGTGAGCCAGATG |
|  |  |  | R:TGTGTGTGTCGTCAGCTTCG |
| *Complement c5* (*c5*) | XM014174798.1 | 121 | F:AAGGCCAGTTGCAGTTCTGT |
|  |  |  | R:CCTGGGAATCCAAAGGGTAT |
| *4-hydroxyphenylpyruvate dioxygenase* (*hpd*) | NM001140426.1 | 184 | F:TTGATGAAGCATGGGGATGGG |
|  |  |  | R:TGAGAGTGTGTGTTGTATCGCC |
| *Elongation factor 1 alpha* (*ef1α)* | BT072490.1 | 88 | F:GCTGTGCGTGACATGAGG |
|  |  |  | R:ACTTTGTGACCTTGCCGC |
| *Interferon γ* (*ifnγ*) | AY795563 | 159 | F:CTAAAGAAGGACAACCGCAG |
|  |  |  | R:CACCGTTAGAGGGAGAAATG |
| *Integrator complex subunit 7* (*ints7*) | XM014204660.1 | 175 | F:ACAACCAGCAGCAGCAACA |
|  |  |  | R:GCTCCAGTCCAGTCTTTTCAAA |
| *Leptin* | FJ830677.1 | 101 | F:CTCCTGTTGTCCTCTCTGT |
|  |  |  | R:ATGGTTTGAGCAAGGTCTTT |
| *Pyruvate dehydrogenase kinase isozyme 2* (*pdk2*) | BT059601.1 | 151 | F:AAGGTGATGGATAGGGGTG |
|  |  |  | R:AGAGGCGTGAAATGGGA |
| *Solute carrier family 13 member 3-like* (*slc13a3*) | BT058859.1 | 130 | F:ACAGGACGAAAGAGCACAA |
|  |  |  | R:CAGGAGAACGGCAAACAAAA |
